# Engrailed-2 (EN2) – a novel biomarker in epithelial ovarian cancer

**DOI:** 10.1186/s12885-018-4816-5

**Published:** 2018-10-03

**Authors:** Sophie Elena McGrath, Nicola Annels, Thumuluru K. Madhuri, Anil Tailor, Simon A. Butler-Manuel, Richard Morgan, Hardev Pandha, Agnieszka Michael

**Affiliations:** 1 Oncology, School of Biosciences and Medicine, FHMS, University of Surrey, Leggett Building, Daphne Jackson Road, Guildford, GU2 7WG UK; 2 Royal Surrey County Hospital, Egerton Road, Guildford, GU2 7XX UK; 3 Present Address: The Royal Marsden NHS Foundation Trust, Downs Rd, Sutton, London SM2 5PT UK; 4 Present Address: The Institute of Cancer Therapeutics, University of Bradford, Bradford, West Yorkshire BD7 1DP UK

**Keywords:** Epithelial ovarian cancer, Biomarker, Engrailed-2, Serous, Platinum-resistant

## Abstract

**Background:**

Epithelial ovarian cancer is a common malignancy, with no clinically approved diagnostic biomarker. Engrailed-2 (EN2) is a homeodomain-containing transcription factor, essential during embryological neural development, which is dysregulated in several cancer types. We evaluated the expression of EN2 in Epithelial ovarian cancer, and reviewed its role as a biomarker.

**Methods:**

We evaluated 8 Epithelial ovarian cancer cell lines, along with > 100 surgical specimens from the Royal Surrey County Hospital (2009–2014). In total, 108 tumours and 5 normal tissue specimens were collected. *En2* mRNA was evaluated by semi-quantitative RT-PCR. Histological sub-type, and platinum-sensitive/−resistant status were compared. Protein expression was assessed in cell lines (immunofluorescence), and in > 150 tumours (immunohistochemistry).

**Results:**

*En2* mRNA expression was elevated in serous ovarian tumours compared with normal ovary (*p* < 0.001), particularly in high-grade serous ovarian cancer (*p* < 0.0001) and in platinum-resistant tumours (*p* = 0.0232). Median Overall Survival and Progression-free Survival were reduced with high *En2* expression (OS = 28 vs 42 months, *p* = 0.0329; PFS = 8 vs 27 months; *p* = 0.0004). Positive cytoplasmic EN2 staining was demonstrated in 78% of Epithelial ovarian cancers, with absence in normal ovary. EN2 positive high-grade serous ovarian cancer patients had a shorter PFS (10 vs 17.5 months; *p* = 0.0103).

**Conclusion:**

The EN2 transcription factor is a novel ovarian cancer biomarker. It demonstrates prognostic value, correlating with worse Overall Survival and Progression-free Survival. It is hoped that further work will validate its use as a biomarker, and provide insight into the role of EN2 in the development, progression and spread of ovarian cancer.

## Background

Epithelial ovarian cancer (EOC) accounts for around 3% of all female cancers, but is the most lethal of the female reproductive tract cancers due to its predominantly advanced stage at diagnosis.

The symptoms of ovarian cancer are often very vague and may be misinterpreted as those of more common, benign conditions. As these symptoms are mostly related to the pressure effects of the growing tumour mass, they may only present when the primary mass is already very large, or when the disease has disseminated to the peritoneum*.* The only clinically utilised biomarker for EOC is CA125, but this has not been approved for use in diagnosis as it may be elevated in benign gynaecological conditions such as endometriosis, and hence has a relatively low sensitivity of 50–62% for early stage disease, although this rises to 90% in advanced stage ovarian cancer [1–3]. CA125 level after the first cycle of chemotherapy and time to normalisation after the start of chemotherapy can be useful as prognostic biomarkers [4, 5].

Many women with advanced EOC at diagnosis will experience a good initial response to platinum-based chemotherapy and surgery, and may have a prolonged disease-free interval, but then often relapse with either local or distant disease. Although relapsed disease can often be controlled with repeated platinum-based chemotherapy regimens, the progression-free relapse time interval will eventually become less than 6 months, indicating that the tumours have developed resistance to platinum.

There is a great deal of interest in biomarker research in EOC especially for early diagnosis as the disease is usually diagnosed at a late stage leading to a 5-year survival rate of only 30% for advanced-stage disease [6, 7]. In addition, there is increasing research into prognostic and treatment response biomarkers, especially focussing on the identification of molecular signatures that may indicate the early development of platinum-resistant disease. The search for novel clinical biomarkers in EOC has included analysis of various homeobox genes and their protein products. The expression of *HOXA9, HOXA10* and *HOXA11* confers a serous, endometrioid and mucinous phenotype respectively [8]. Kelly et al*.* also demonstrated that high expression of HOXA13, B6, C13, D1 and D13 were predictive of poor clinical outcome in EOC [9]. More recently, Miller et al demonstrated that HOXA4/HOXB3 overexpression in high-grade serous ovarian cancer, correlated with a significantly shorter PFS, most likely as a result of reduced platinum sensitivity [10]. PAX8, is consistently over-expressed in high grade serous ovarian carcinomas but negative in breast adenocarcinoma so is often used by the pathologist to help determine the origin of certain pelvic serous tumours, especially if the primary tumour source is not always clearly evident [11–13].

Engrailed-2 (EN2) is a homeobox protein, essential during embryological neural development and typically only expressed in the normal adult Purkinje neurons and kidney tubular epithelial cells. EN2 is dysregulated in several cancer types, but at present there is no published data on its presence in EOC. *EN2* mRNA over-expression has been demonstrated in breast and prostatic adenocarcinoma whilst it is not expressed in normal prostate and breast tissue, or in benign disease [14, 15]. Secretion of EN2 protein into the prostatic ductal lumen was also noted, prompting further investigation of EN2 as a biomarker in prostate cancer. The presence of EN2 in urine was highly predictive of prostate cancer, with a sensitivity of 66% and specificity of 88.2% [15]. A linear relationship between urinary EN2 and prostate cancer volume has been demonstrated, along with a relationship between urinary EN2 and tumour stage [16, 17]. Elevated levels of urinary EN2 have also been detected in patients with urothelial bladder cancer, with an overall sensitivity of 82% and specificity of 75% [18]. A study of 226 patients with high grade urothelial bladder cancer showed that EN2 expression in cancer was strongly predictive of poor prognosis (manuscript in preparation).

Based on the paucity of diagnostic and prognostic biomarkers in EOC, and the growing body of evidence that EN2 has a role in other epithelial cancers, we evaluated *En2* gene and EN2 protein expression in a broad selection of EOC cell lines and human tumour tissues and examined its relationship with clinical characteristics. We hypothesised that over-expression of *En2* mRNA and EN2 protein in human EOC tumours, may prove useful as a diagnostic, prognostic or treatment response biomarker, helping to influence treatment decisions, and ultimately improve overall survival rates.

## Methods

### Patients and controls

This study was approved by the London - Brighton & Sussex Research Ethics Committee (REC no: 09/H1103/50) and conducted between 2009 and 2014. Fresh ovarian tissue was obtained from patients at The Royal Surrey County Hospital, at the time of their primary surgery and then stored in RNAlater™ (Sigma-Aldrich, UK) at − 20 °C. In total 108 tumours and 5 normal tissue specimens were collected. Normal human ovary RNA was purchased from OriGene Technologies Inc., USA.

A separate cohort of 90 pre-cut slides from formalin-fixed, paraffin embedded ovarian tumours were obtained from The Royal Surrey County Hospital, along with normal ovary and kidney slides as controls.

### Human cell lines

All cell lines were obtained from the American Type Culture Collection (ATCC, USA), except for PEO1, PEO14 and PEO23 [Health Protection Agency (HPA, UK)], PEO4, PEA1 and PEA2 (donated by Professor Hani Gabra at Imperial College, University of London), and Fibroblasts (donated by the University of Birmingham). All of the human cell lines were adherent lines and were maintained according to the supplied protocol. In the matched pairs, the first set of cell lines (PEO1, PEO14, PEA1) were derived following the patients’ diagnosis, while the second set (PEO4, PEO23, PEA2) were derived following the onset of acquired clinical platinum resistance. The normal human skin fibroblast cell line was used as a negative control and the melanoma cell line, A375M, was used as a positive control.

### RNA extraction & cDNA synthesis from cell lines & human tumours

Total RNA was extracted from cell lines and homogenised tumours using the RNeasy® Plus Mini Kit (Qiagen, UK) according to the manufacturer’s instructions. Extracted RNA was eluted in RNase-free water and quantified using a Nanodrop ND-1000 Spectrophotometer. cDNA was synthesised from total RNA using the Cloned AMV First Strand cDNA Synthesis Kit (Invitrogen, UK) following the manufacturer’s protocol, using 1 μg of RNA. cDNA samples were stored at − 20 °C (10 ng/μl).

### Semi-quantitative RT-PCR

Semi-quantitative RT-PCR was performed using the Stratagene MX3005P Real Time PCR machine (Agilent Technologies, UK), measuring PCR product accumulation during the exponential phase of the reaction by SYBR green fluorescence (SYBR® Green JumpStart™ Taq ReadyMix™ Kit, Sigma-Aldrich, UK). Reaction conditions were 1 cycle of 94 °C for 10 min, 40 cycles of 30 s at 94 °C, and 1 min at 60 °C and 30 s at 72 °C. The forward and reverse primers for *En2* were 5′ GAACCCGAACAAAGAGGACA 3′ and 5′ CGCTTGTTCTGGAACCAAAT 3′, and for *ß-actin* were 5′ ATGTA CCCTGGCATTGCCGACA 3′ and 5′ GACTCGTCATACTCCTGCTTGT 3′. Relative expression was calculated using the ∆C_T_ comparative method (2^-∆Ct^) [15, 18], and expression is shown relative to *ß-actin* (× 100,000).

### Enzymatic immunofluorescent staining on cell lines

Cell lines were incubated for monolayer growth in 8-chambered polystyrene culture treated glass slides (BD Biosciences, UK) with appropriate media. Staining was compared in the absence of primary antibody, and in non-permeabilised and permeabilised cells (0.2% Triton X-100 (Sigma-Aldrich, UK). Cells were incubated in 5 μg/ml Wheat-Germ Agglutinin cell membrane stain (Invitrogen, UK) for 10 min at 37 °C, before fixation with warm 4% paraformaldehyde, and blocking with 4% horse serum. Cells were incubated overnight in polyclonal goat anti-EN2 antibody (Abcam, UK) diluted 1:100 in 1% BSA (Sigma-Aldrich, UK) in PBS or in 1% BSA in PBS alone. This primary antibody has been used within the department for immunofluorescence work in multiple cells lines derived from varying tumour types, with consistent results. The secondary antibody Alexa Fluor 488 donkey anti-goat IgG (Invitrogen, UK) diluted 1:200 in 1% BSA/PBS was added, along with the nuclear stain TO-PRO-3 (Life Technologies, UK) diluted 1:400, at room temperature for 1 h. The slides were mounted and visualised using a Zeiss LSM 510 confocal laser scanning microscope, and a Plan-Apochromat 40× oil immersion objective. Images were recorded and analysed using ZEN 2009 capture software.

### Enzymatic immunohistochemistry on patient slides and tissue arrays

Following the deparaffinisation and rehydration of slides, the heat mediated antigen retrieval method was utilised, using citrate buffer (10 mM, pH 6.0). Polyclonal rabbit anti-EN2 antibody (LS-B3477, LifeSpan Biosciences, Seattle, USA,) was diluted 1:20 in 1% BSA in PBS and applied overnight. 1% BSA in PBS alone was added to control slides. The bound primary antibody was detected using the R.T.U. VECTASTAIN ABC Kit (Vector Laboratories, USA) followed by DAB detection. Slides were counterstained with Haematoxylin and mounted. The Royal Surrey County Hospital Pathology Department performed automated Haematoxylin & Eosin (H&E) staining on adjacent sections.

Slides were scored by two independent investigators for immuno-intensity of EN2 staining: 0, negative; 1, weak; 2, moderate; 3, strong. The percentage of immuno-positive cells was also recorded and scored from 1 to 4. The product of immuno-intensity and immuno-positivity ranged from 0 to 12, with 0–4 representing EN2 negative staining, and 5–12 representing EN2 positive staining [19]. All slides were reviewed by an independent gynaecological pathologist.

### Statistical analysis

The GraphPad Prism software package was used to construct graphs and for statistical calculations. The significance level for all tests was set at 5%. The following symbols were used in diagrams/figures to denote levels of significance: -.* = *p* < 0.05; ** = *p* < 0.01; *** = *p* < 0.001; **** = *p* < 0.0001.

The one-way ANOVA with Bonferroni correction was performed for *En2* mRNA expression cell line data, using the mean CT value relative to *ß-actin* [20–22]. For the different histological sub-types of human tumour tissue, the non-parametric Kruskal Wallis test with Dunn’s correction was used. For comparison of two groups, the non-parametric Mann-Whitney *U*-test was used.

Comparative analysis of EN2 positive and negative tumour sections and tissue cores was conducted using the Chi-squared test [23].

All survival analyses were performed using the Log-rank (Mantel Cox) test.

## Results

### EN2 expression in epithelial ovarian cancer cell lines

RNA was extracted from cultures of 8 serous EOC cell lines, with the platinum-sensitivity status recorded for each of these. PEO1 represented a cell line derived from a platinum-sensitive, serous ovarian carcinoma. The PEO4 cell line was derived from the same patient, once they had developed platinum-resistant disease. Similarly, PEO14 and PEO23, and PEA1 and PEA2, were paired cell lines derived from the same patient before and after developing platinum-resistant disease.

**Fig. 1:**
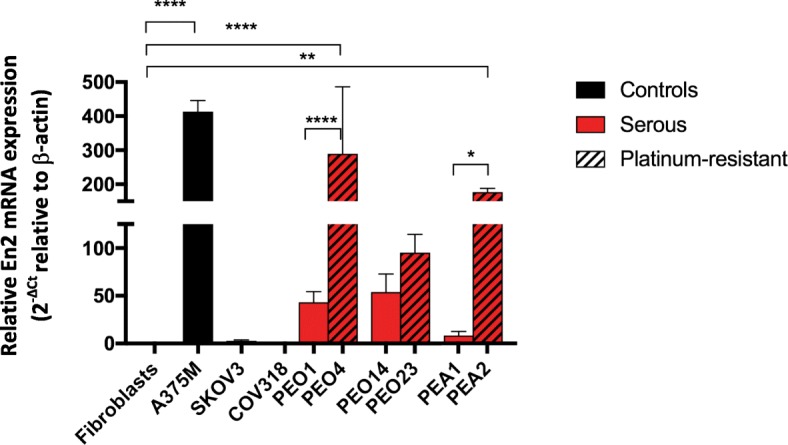
*En2* mRNA expression in EOC cell lines and controls. Eight ovarian cancer cell lines were analysed by quantitative RT-PCR. Fibroblasts were used as a negative control and the A375M cell line as a positive control. En2 mRNA expression is shown relative to β-actin (× 100,000), using the ∆C_T_ comparative method (2^-∆Ct^). Error bars represent the SD (*n* = 3) (* = *p* < 0.05; ** = *p* < 0.01; **** = *p* < 0.0001)

Evaluation of the EOC cell lines revealed *En2* expression in the majority of cell lines, but to a varying degree (Fig. 1). The serous EOC lines PEO4 and PEA2 demonstrated significantly higher *En2* expression than fibroblasts (*p* < 0.0001 and *p* < 0.01 respectively). The platinum-sensitive/resistant paired serous cell lines all showed elevated *En2* expression, however there was a significant increase in the platinum-resistant lines, compared with their platinum-sensitive pairings. This was most notable with the PEO1/PEO4 (*p* < 0.0001) and PEA1/PEA2 (*p* < 0.05) paired cell lines.

**Fig. 2:**
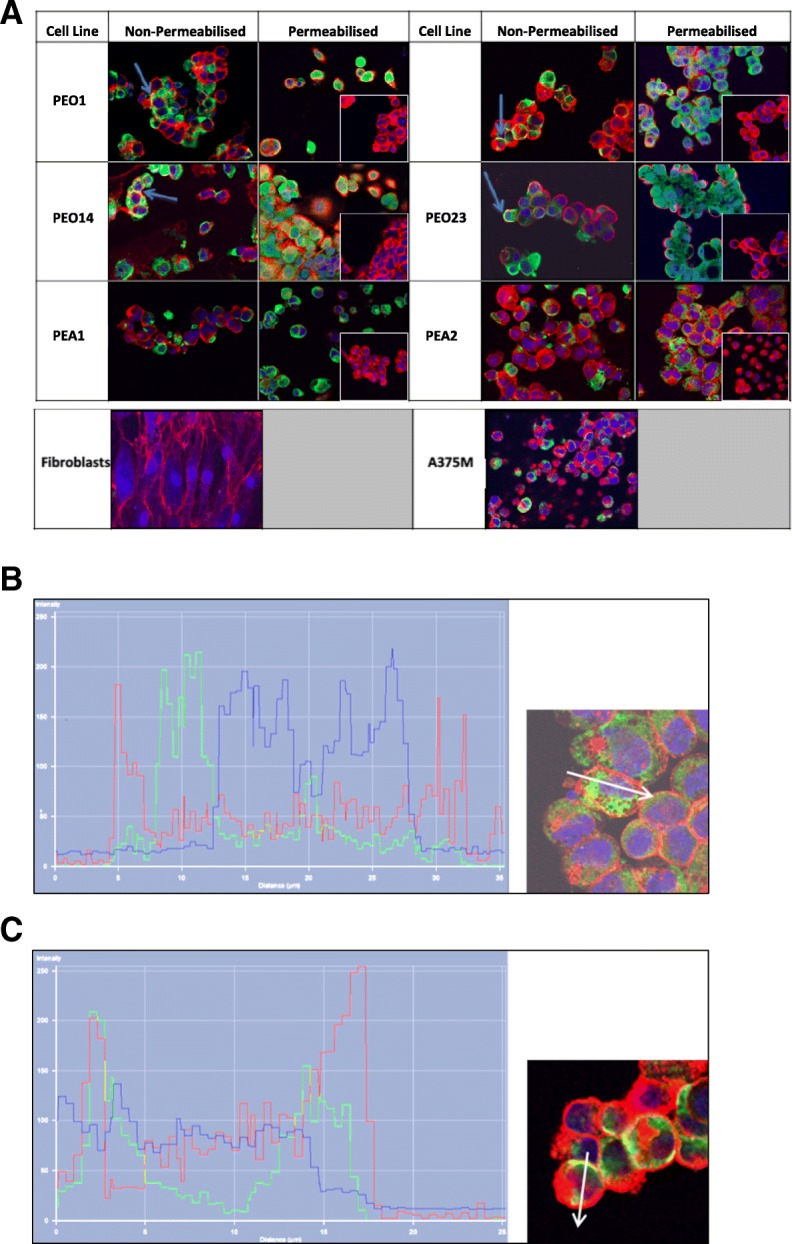
EN2 expression via immunofluorescent staining of platinum-sensitive/−resistant paired serous ovarian cancer cell lines and controls. (**a**) EN2 fluorescent staining (green) was present in the cytoplasm of the serous cell lines, particularly evident in those that had been permeabilised. There was no EN2 staining evident in the permeabilised negative control of each cell line, where only secondary antibody was added (inset). There was no EN2 staining evident in the fibroblast cell line, whilst there was strong cytoplasmic staining in the melanoma A375M positive control cell line. TOPRO staining (blue) identified the cell nucleus and WGA staining (red) identified the cell membrane. Co-localisation of cell membrane WGA and EN2 protein staining (yellow) can be seen in some of the non-permeabilised cells (blue arrows). (**b**) Intensity profiles for each label along the length of the white arrow are shown in a PEA2 cell, and a PEO4 cell (**c**)**,** demonstrating the absence of EN2 in the nucleus, but close association with the cell membrane. The coloured peaks represent the cell membrane, nucleus, presence of EN2, and co-localisation of EN2 with the cell membrane, as detailed above

For assessment of EN2 protein in EOC cell lines, enzymatic immunofluorescent staining was performed. This analysis demonstrated that EN2 protein was present in all of the EOC cell lines tested, in particular the paired platinum sensitive/insensitive cell lines, and the A375M melanoma cells, whilst it was not detectable in the normal skin fibroblast cell line (Fig. 2a). EN2 appeared to be predominantly expressed within the cytoplasm of the cancer cell lines, especially after the cells were permeabilised. There was no co-localisation of nuclear and EN2 protein staining to suggest the presence of nuclear EN2 in any of the cell lines stained (Fig. 2b). Co-localisation of cell membrane and EN2 protein staining suggested that EN2 is located in close proximity to the cell membrane (Fig. 2c).

### EN2 expression in human ovarian tissue

We subsequently analysed 108 tumour samples, representing a variety of EOC histological sub-types (Table 1).

**Table 1:** Summary data from the human ovarian tumours Cohort 1

Histology	*N*	Mean age (range)	Median Grade	Median Stage	Surgery (Number of patients)		Platinum Status (Number of patients)		Median PFS	Median OS
					Primary	Interval	Sensitive	Resistant		
Pelvic serous carcinoma	75	65.28 (40–86)	3	3	22	53	56	17	10	31
Serous adenocarcinoma	58	65.60 (42–86)	3	3	20	38	43	14	10	32
Primary peritoneal adenocarcinoma	17	64.47 (40–74)	3	3	2	15	13	3	10	34
Endometrioid adenocarcinoma	8	61.63 (45–84)	3	3	7	1	5	2	11	30.5
Mucinous adenocarcinoma	2	54 (48–60)	3	3	1	1	0	1	1	6.5
Clear cell carcinoma	4	49(40–62)	3	3	4	0	1	2	1	14
MMMT	6	72.50 (62–90)	3	3	5	1	0	3	1.5	5
Borderline epithelial tumour	8	56 (36–69)	–	–	8	0	–	–	–	–
Benign tumour	5	55.2 (48–68)	–	–	–	–	–	–	–	–
Normal ovary	5	67.58 (54–84)	–	–	–	–	–	–	–	–

**Fig. 3:**
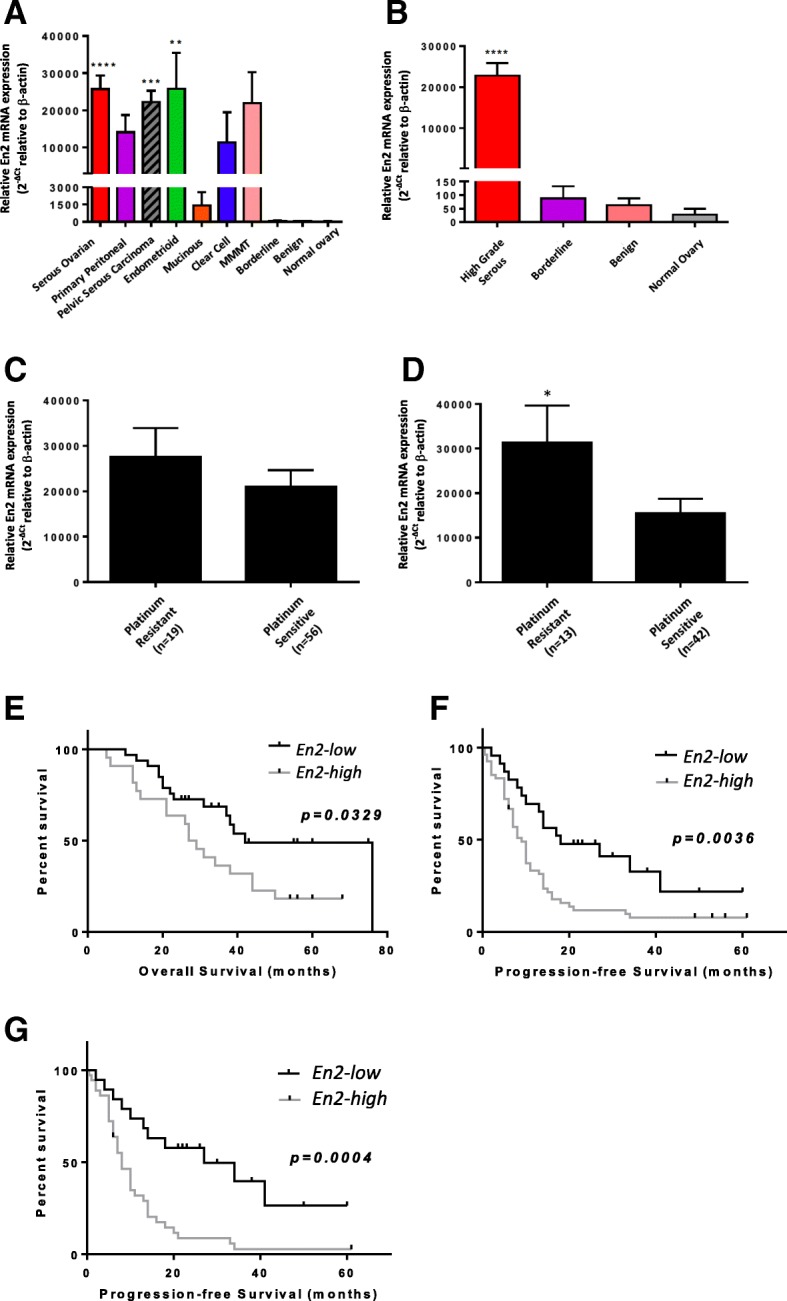
*En2* mRNA expression is elevated in high-grade serous tumours, with higher expression conveying poor prognosis. Malignant, borderline, benign and normal tumours were analysed, with mean En2 mRNA expression for each histological sub-type (**a**), or high grade serous tumours (HGSOCs) and non-malignant specimens (**b**), shown. Follow-up data was studied to determine if the patient had platinum-resistant (PFS < 6 months) or platinum-sensitive (PFS ≥ 6 months) disease. En2 mRNA expression is shown for all HGSOCs (**c**), and interval surgery HGSOCs versus platinum status (**d**)(*p* = 0.0232). Survival curves compare high versus low En2 expression for (**e**) overall survival in interval surgery HGSOCs (median OS = 28 vs. 42 months; *p* = 0.0329), (**f**) progression-free survival in all HGSOC specimens (median PFS = 9 vs. 18 months; p = 0,0036), and (**g**) progression-free survival in interval surgery HGSOCs (median PFS = 8 vs. 27 months; *p* = 0.0004). `*En2* mRNA expression is shown relative to *β-actin* (× 100,000), using the ∆C_T_ comparative method (2^-∆Ct^). Error bars represent the SE; (* = *p* < 0.05; ** = *p* < 0.01; *** = *p* < 0.001; **** = *p* < 0.0001)

All serous ovarian and peritoneal tumours were grouped together as “pelvic serous carcinoma”, a term suggested by Nik et al. [24]. The relative expression of *En2* in normal ovary was very low in comparison to that of the malignant tumour samples. Although higher than the normal specimens, the *En2* expression in benign and borderline epithelial tumours was also much lower than the malignant tumours. *En2* expression in the serous ovarian group was significantly elevated in comparison to the normal specimen group (*p* < 0.001), namely > 1200-fold higher, and this was maintained when the serous ovarian and peritoneal groups were combined as the “pelvic serous carcinoma” group (*p* < 0.005) (Fig. 3a). High-grade serous ovarian carcinomas (HGSOC) are the most common histological presentation in the clinic, and represented 97% of the pelvic serous tumours within this data set. *En2* expression in the HGSOC group was again significantly elevated in comparison to the normal specimen group (*p* < 0.0001) (Fig. 3b).

Platinum-based chemotherapy treatment data was available for 75 of the high-grade serous epithelial tumours, including progression-free survival, which allowed us to determine the platinum-sensitivity-status of the tumours as defined by Blackledge [25]. *En2* expression was higher in platinum-resistant tumours compared to those that were platinum-sensitive, particularly when considering those patients who had already received some chemotherapy prior to surgery, i.e. the interval surgery cohort (Fig. 3c and d); *p* = 0.0232).

Survival analyses of the HGSOCs were performed based on low versus high *En2* mRNA expression. As *En2* expression had not previously been evaluated in ovarian cancer, we had little guide as to what constituted low or high expression, so we defined low expression as a CT value relative to *ß-actin* < 1000, and high expression as > 1000. This took into consideration, the fact that all borderline, benign and normal ovarian tissue had mean CT values relative to *ß-actin* < 100. When analysing the HGSOC “interval surgery specimens”, both the median overall survival (OS) and median progression-free survival (PFS) in the high *En2* group were significantly lower than in the low *En2* group (28 vs 42 months for OS; *p* = 0.0329 (Fig. 3e), and 9 vs 18 months for PFS; *p* = 0.0036 (Fig. 3f)). This difference in PFS was even more pronounced when considering only the interval surgical HGSOCs (8 vs 27 months; *p* = 0.0004 (Fig. 3g)).

**Fig. 4:**
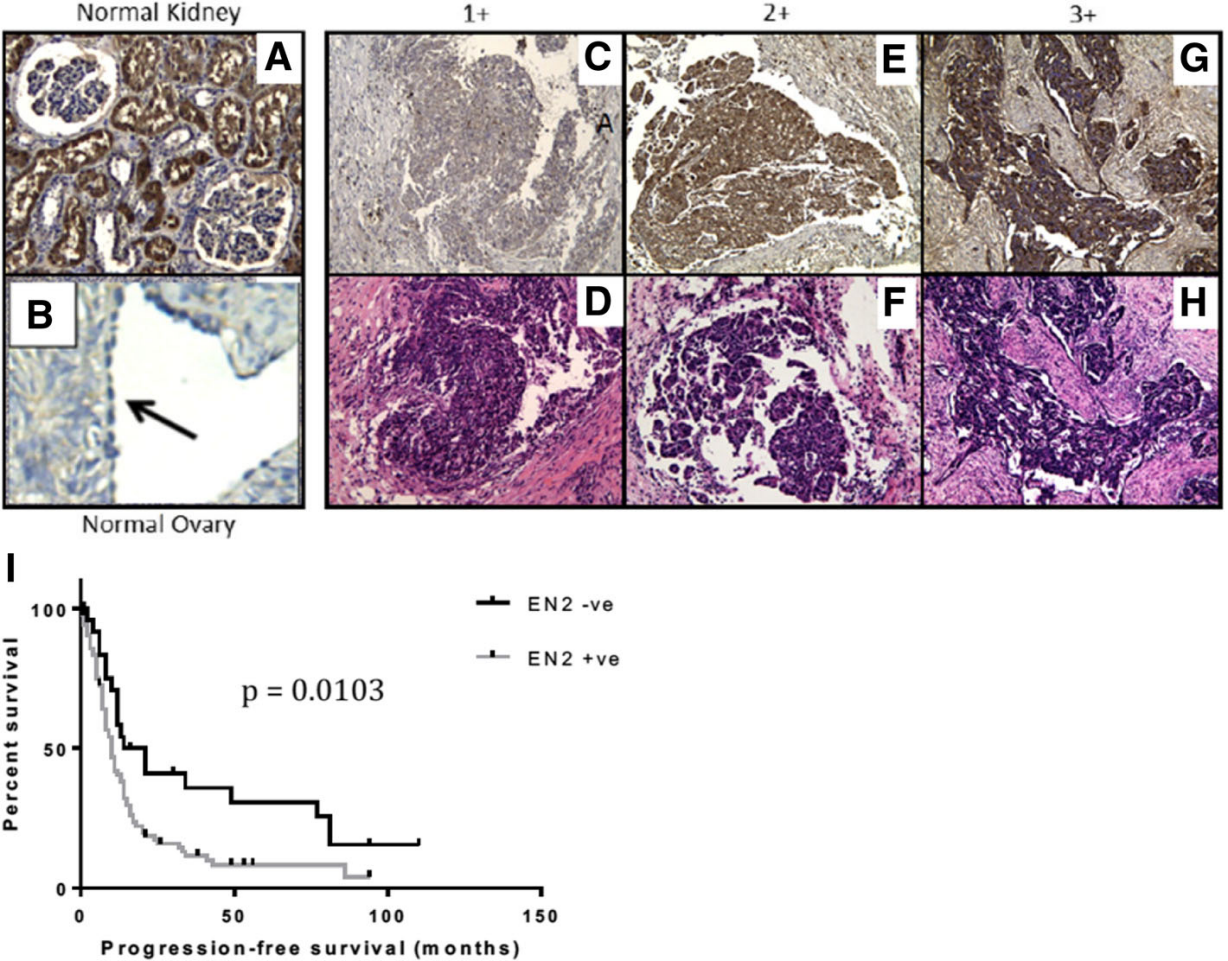
EN2 protein expression in human ovarian tissue conveys poor prognosis. Enzymatic immunohistochemistry examples of normal kidney (**a**), normal ovary with surface epithelium (**b**) and ovarian adenocarcinoma stained for EN2 (**c**, **e**, **g**), or adjacent sections stained for H&E (**d**, **f**, **h**), at 10× magnification. The assigned staining intensity (1–3+) is indicated. EN2 staining (brown) is present in the cytoplasm, but not in the nucleus, and is not seen in the surrounding stroma. On the H&E stained sections, pink staining represents cytoplasm and blue staining represents the nucleus. (**i**) Survival curves for EN2 negative versus EN2 positive protein expression in high-grade serous ovarian tumours are shown (EN2 -ve = 0–4 IHC score; EN2 + ve = 5–12 IHC score). The median survival was significantly lower for all HGSOCs with EN2 + ve staining (10 months) versus those with EN2 –ve staining (17.5 months) (*p* = 0.0103)

The prevalence and expression pattern of EN2 was also assessed at the protein level in two separate cohorts of patient tumours which enabled the combined analysis of a large number of EOC specimens (111 HGSOCs in total). The summary demographic data for these cohorts are shown in Tables 1 and 2. Examples of the EN2 protein expression in human ovarian tissue are shown in Fig. 4.

**Table 2:** Summary data from the human ovarian tumours Cohort 2

Histology	*N*	Mean age (range)	Median Grade	Median Stage	Surgery (Number of patients)		Platinum Status (Number of patients)		Median PFS	Median OS
					Primary	Interval	Sensitive	Resistant		
Pelvic serous carcinoma	44	60.18 (25–81)	3	3	30	14	33	5	13.5	41
Endometrioid adenocarcinoma	12	63 (44–84)	2	1	12	0	10	0	97	105
Mucinous adenocarcinoma	6	64.67 (42–84)	3	3	6	0	1	2	1	9.5
Clear cell carcinoma	1	58	3	3	1	0	–	–	1	1
MMMT	3	71.67 (69–75)	3	3	2	1	–	2	1.5	5
Borderline epithelial tumour	17	54.18 (25–89)	–	–	17	0	–	–	–	–
Benign tumour	7	64.86 (46–80)	–	–	7	0	–	–	–	–

Positive cytoplasmic EN2 staining was demonstrated in 78% of EOCs and over 65% of borderline tumours, whilst there was no staining in the majority of benign tumours and in all normal ovary specimens. Comparative analysis of the tumour sections confirmed a significant difference between EOC and borderline tumours compared to benign and normal samples (Table 3). Patients with HGSOC tumours staining positive for EN2 had a significantly shorter median PFS than those with EN2 negative tumours (10 vs 17.5 months; *p* = 0.0103) (Fig. 4i). This difference is similar to that observed for *En2* mRNA expression. There was no significant difference in OS between the groups.

**Table 3:**
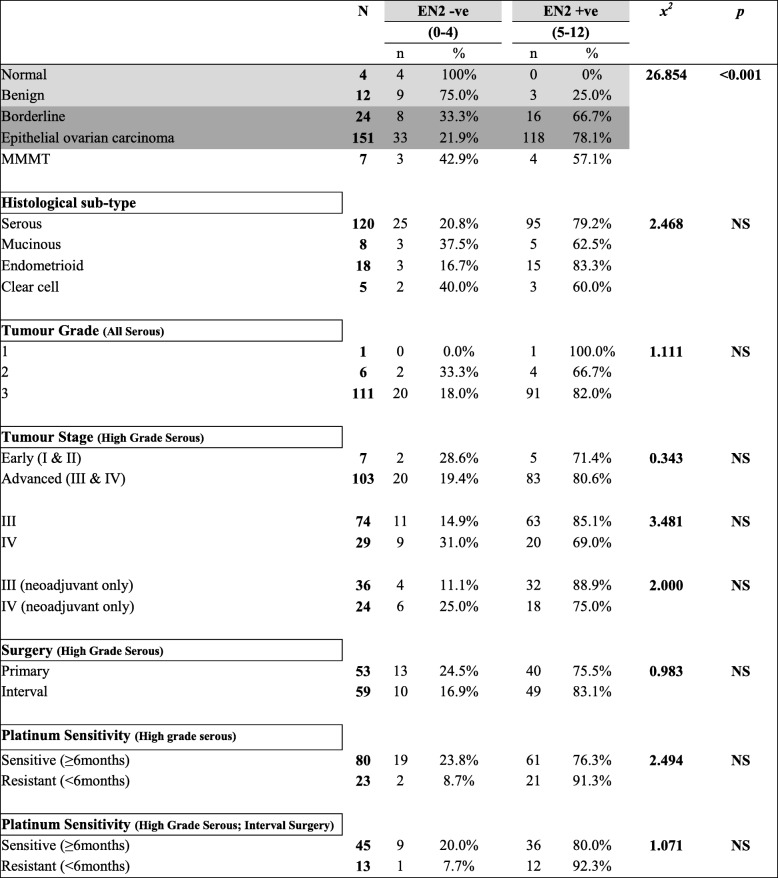
EN2 protein expression in human ovarian tissue (Cohorts 1 and 2)

The product of the EN2 staining intensity score (0–3+) and the percentage of positively stained cells score (0–4) was calculated. The resultant score of 0–4 represented EN2 negative staining, and 5–12 represented EN2 positive staining. Comparative analysis of the tumour sections demonstrated a significant difference between the EOC and borderline tumours, compared with the benign and normal tissues (*x*^*2*^ = 26.854, *p* < 0.001)

Confirmatory immunohistochemical staining of three ovarian cancer tissue arrays was performed to confirm the findings in our patient cohorts, and again demonstrated high levels of EN2 protein staining in EOC cores, particularly the serous histological sub-type (data not shown). As with our patient analysis, there was no EN2 staining in the epithelial or stromal components of normal ovary or normal adjacent ovary tissue.

## Discussion

Despite significant advances in the medical management of solid tumours over the past decade, there has been little impact on the survival of advanced EOC. CA125 remains the only approved serum biomarker in ovarian cancer, however its routine use is restricted to monitoring ovarian cancer progression and treatment response. Other mucin-related glycoproteins such as MUC1 (CA15–3), HE4 and mesothelin have been extensively studied, alone or in combination with CA125, but still lack the desired high sensitivity and specificity necessary for a gold-standard clinical biomarker [26–29].

Many developmental homeodomain-containing transcription factors are aberrantly expressed in cancer and have been shown to promote cancer development, progression, recurrence or development of drug resistance [30, 31]. *EN2* encodes a transcription factor that plays an important role in embryonic development, but appears to have limited function in the normal adult nervous system [32, 33]. EN2 over-expression has been identified in a number of adult human cancers, namely breast, prostate, and bladder, and appears to have a functional role in tumour development. In EOC, the homeobox transcription factors HOXA13, B6, C13, D1 and D13 may be useful as prognostic biomarkers in EOC [9], and PAX8 is regularly used as a contributory diagnostic biomarker in EOC [11–13]. Preliminary work on cell lines and tissue arrays suggested that EN2 may be present in ovarian cancer [34]. To test its role as a diagnostic or prognostic biomarker, *En2* RNA and protein expression in EOC cell lines and tumour tissue was characterised and its relationship with clinico-pathological parameters was assessed.

We demonstrated *En2* mRNA and EN2 protein over-expression in a number of EOC representative cell lines, and observed significantly higher *En2* mRNA expression in platinum-resistant compared with paired platinum-sensitive cells. However this was not seen in other examples of serous and endometrioid platinum-resistant cell lines. Some of these discrepancies may be as a result of inaccurate histological sub-type labelling. In a recent publication it was reported that the well-used SKOV3 cell line, previously thought to represent a high grade serous ovarian cancer, is actually more in keeping with an endometrioid pathology on molecular analysis [35].

EN2 protein was detectable via immunohistochemistry in all of the cell lines to a varying degree but when quantified its expression was found to be higher in platinum-sensitive compared to platinum-resistant paired cell lines. Although cell line work can be very informative and guide on-going work, it is not always fully representative of what occurs in the human tumour in vivo, especially if derived from ascitic fluid or metastatic tumour deposits rather than the primary tumour. Similar work on human tumour samples was vital to draw more comprehensive conclusions regarding EN2 expression in EOC.

*En2* mRNA expression in ovarian tissue could provide useful clinical information, although its use as a diagnostic marker of EOC would seem to be limited. EN2 protein levels in high-grade serous tumours was much higher than in normal ovarian specimens, and very low in non-invasive borderline and benign serous ovarian tumours. It may also help to provide an early indication of whether the original tumour derives from the ovary or peritoneum, given that *En2* mRNA levels for the latter group were lower. At present, the primary source of the cancer is often not confirmed until primary or interval-debulking surgery has been completed. EN2 protein staining was also detected in the majority of EOC and borderline tumour specimens, and was negative in all normal ovary specimens, and in most benign tumours. However the intensity of staining did not reliably distinguish between the histological sub-type, grade, or stage of disease.

Our findings indicate that *En2* mRNA expression could act as a prognostic biomarker in interval debulking surgery in high-grade serous EOC tissue where an elevated level predicted a shorter PFS. Elevated *En2* mRNA levels also suggested a shorter OS, further supporting its possible use as a prognostic biomarker, although 40% of the OS data remained censored at the time of analysis. There is growing data to suggest that a number of homeobox transcription factors may play a prognostic role in serous ovarian cancer [9, 10]. Therefore combining these HOX genes with *EN2* expression, may also prove useful as a scoring system for prognostic risk stratification.

EN2 protein analysis of interval debulking surgery high-grade serous tumour specimens also demonstrated its possible utility as a prognostic biomarker. Patients with EN2-positive tumours had a shorter PFS, with durations in keeping with the mRNA data. However, there were no significant differences in OS. Given that interval debulking surgery with pathological analysis is commonplace in the management of high-grade serous EOC, evaluation of *En2* mRNA or EN2 protein expression could help the oncologist to devise a more individualised post-operative treatment regimen. An elevated *En2* tumour mRNA level at interval debulking surgery in high-grade serous EOC patients was associated with early disease relapse, suggesting resistance to platinum chemotherapy. In combination with the surgeon’s assessment of the amount of residual disease at debulking surgery, the level of *En2* mRNA could be used to assess treatment response, and guide the oncologist in deciding whether to prescribe additional cycles of post-operative chemotherapy, or to change the combination of drugs.

Increased *En2* mRNA and EN2 protein expression were identified in platinum-resistant EOC cell line models and human tissue samples compared with platinum-sensitive cases. In the case of the paired EOC cell lines, this suggested that increased *En2* expression may directly influence the disease progression or the development of platinum-resistance. We demonstrated that EN2 protein was located in the cytoplasm of EOC cell lines and tumour tissue, and not the nucleus, as seen in normal adult Purkinje neurones. This may suggest that it has a role in translational, rather than transcriptional regulation, possibly through a similar mechanism to that demonstrated in neurons of the developing nervous system [36]. In certain cell lines, it was visualised in association with the cell membrane, which may relate to cellular secretion or uptake.

An ideal early diagnostic biomarker would be a serological or urine-based biomarker which enables minimally invasive collection and low cost. Unfortunately, EN2 has proven difficult to isolate in blood (personal communication with Prof Richard Morgan), and although detectable in urine in known ovarian cancer sufferers, additional work is required to assess its utility.

## Conclusion

This original research presents the Engrailed-2 transcription factor as a novel ovarian cancer biomarker. EN2 demonstrates prognostic value, particularly in interval debulking high-grade serous EOC tissue, correlating with worse overall and progression-free survival. Clearly, there is still an unmet need for robust diagnostic, as well as prognostic biomarkers in EOC. Therefore, it is hoped that further work will validate the use of EN2 as a such a biomarker, and provide further insight into the role of EN2 in the development, progression and spread of ovarian cancer.

## Abbreviations

∆C_T_: Change in cycle threshold
ANOVA: Analysis of Variance
BSA: Bovine Serum Albumin
CA125: Carcinoma Antigen 125
cDNA: Complementary Deoxyribonucleic Acid
CT: Cycle threshold
DAB: 3,3′-diaminobenzidine
DNA: Deoxyribonucleic Acid
*En2*: Engrailed 2 gene
EN2: Engrailed 2 protein
EOC: Epithelial Ovarian Cancer
H&E: Haematoxylin and Eosin
HGSOC: High Grade Serous Ovarian Cancer
*HOX*: Homeobox gene
IHC: Immunohistochemistry
mRNA: Messenger Ribonucleic Acid
OS: Overall Survival
*PAX*: Paired gene
PBS: Phosphate Buffered Saline
PCR: Polymerase Chain Reaction
PFS: Progression-free Survival
REC: Research Ethics Committee
RNA: Ribonucleic Acid
RSCH: The Royal Surrey County Hospital
rt-PCR: Reverse Transcriptase Polymerase Chain Reaction
